# Liver Iron Concentration and Liver Impairment in Relation to Serum IGF-1 Levels in Thalassaemia Major Patients: A Retrospective Study

**DOI:** 10.4084/MJHID.2015.016

**Published:** 2015-02-20

**Authors:** Vincenzo De Sanctis, Ashraf T Soliman, Giancarlo Candini, Christos Kattamis, Giuseppe Raiola, Heba Elsedfy

**Affiliations:** 1 Pediatric and Adolescent Outpatient Clinic, Quisisana Hospital, Ferrara, Italy; 2 Department of Pediatrics, Division of Endocrinology, Alexandria University Children’s Hospital, Alexandria; 3 Department of Medical Physics, St. Anna Hospital, Ferrara, Italy; 4 First Department of Paediatrics, University of Athens, Athens, Greece; 5 Department of Paediatrics, Pugliese-Ciaccio Hospital, Catanzaro, Italy; 6 Department of Pediatrics, Ain Shams University, Cairo, Egypt

**Dear Sir,**

In the postnatal period, the liver remains the main source of circulating insulin-like growth factor 1 (IGF-1) and its synthesis is mainly under the effect of growth hormone (GH). Secretion of IGF-1 is also related to age, gender, genetic factors, nutrition, insulin, and disease conditions^1^. IGF-1 produced in the liver exerts mainly endocrine activity while IGF-1 synthesized by other tissues acts in a para- and/or autocrine manner. IGF-1 is an anabolic hormone that causes a decrease in proteolysis and an increased stimulation of protein production, followed by an increment in muscular mass 1. The most significant expression of IGF-1 deficiency exists in Laron-type dwarfism 1. In thalassaemia major (TM), IGF-1 deficiency has been attributed to chronic anemia and hypoxia, chronic liver disease, iron overload and other associated endocrinopathies, e.g. GH deficiency 2,3.

In a previous report, we found that IGF-1 levels were 2SDs below average values for healthy individuals in 60 out of 120 thalassemic patients (50%) (33 males and 27 females). In these patients endocrine complications and elevation of aminotransferases (ALT) were more common compared to TM patients with IGF-1 > -2SDs^2^. However, the influence of liver iron overload and the severity of chronic liver disease in terms of both grade and stage were not fully elucidated 2. Therefore, we reviewed the available data in 85 β TM patients followed in the last two decades at the Thalassaemia Centre and Quisisana Pediatric and Adolescent Outpatient Clinic of Ferrara. The present report is a part of an ongoing retrospective study identifying the factors responsible of IGF-1 deficiency in TM.

The following clinical and laboratory data were registered: age at first transfusion, height, weight, body mass index (BMI), pubertal status, serum ferritin, creatinine, alanine aminotransferase (ALT), gamma glutamyl transferase (**γ**GT), alkaline phosphatase (ALP), total and direct bilirubin, albumin, prothrombin time (PT), international normalized ratio (INR) and serologic screening assays for hepatitis C virus sero-positivity (HCVab and HCV-RNA). Patients with thalassaemia intermedia, cardiac or renal failure, malnutrition and HIV positivity were excluded from the study.

A total of 20 TM patients underwent liver biopsy at mean age of 26 years (range 19 to 32 years) for persistent increase of liver enzymes (6 months or more). Based on liver ultrasound tests and α-fetoprotein (AFP) levels, neoplastic growth (HCC) was not suspected in any of the patients. Histopathological findings were analyzed following the classical haematoxylin–eosin staining employing a numerical scoring system for the grading (G=0–3) and the stage of fibrosis (S=0–4) according to the METAVIR Cooperative Study Group 4. The degree and cellular distribution of iron stores was assessed using Perls’ Prussian blue stain. The liver iron concentration (LIC) was assayed by atomic absorption spectrophotometry and expressed as mg/g dry weight (dw) 5.

Quantitative estimation of LIC was done in 65 TM patients, between the ages of 15 to 48 years, by Superconducting Quantum Interference Device (SQUID) susceptometry 6. Based on data from the literature in normal people LIC is between 0.4 and 2 mg/g of liver dry weight, while in subjects with iron overload is classified as mild, 2–7, moderate, 7–15 and severe > 15mg Fe/gr dry wt. Patients with LIC level *>* 15 mg/g have increased liver enzyme levels, progression to liver fibrosis and increased risk of premature death.^7^

Serum ferritin was measured at six monthly intervals and the mean serum ferritin in the year before evaluation was recorded. Serum ALT levels were routinely measured prior to monthly transfusions and the annual levels before the evaluation were recorded. IGF-1 was measured using commercial automated immunoassay following the manufacturer’s instructions. The reported analytic sensitivity of this assay was from 6 to 25 ng/ml. Ranges of normal values set at the 2.5th–97.5th percentile in 547 non-hypopituitary, non-acromegalic healthy subjects of both sexes in Italy in three age ranges were: 95.6–366.7 ng/ml for ages 25 to 39 yr, 60.8–297.7 ng/ml for 40 to 59 yr and 34.5–219.8 ng/ml for subjects aged 60 and above^8^.

Characteristics of the studied patients are reported as mean, median, number and range. Fisher’ exact test was used to calculate the probability value for the relationship between two dichotomous variables. A p value < 0.05 was considered significant.

A software program used for the statistical analysis was developed by Dr. Candini (Department of Medical Physics, St. Anna Hospital, Ferrara, Italy) and validated according to Alder and Roesser^9^.

Forty-two females and 43 males TM patients were included in our retrospective survey (mean age at the last observation: 36.6 years; age range: 15.1–53.1 years). Eleven (12.9%; 3 females) had insulin dependent diabetes mellitus. The body mass index (BMI) ranged between 17.4 to 30.8 kg/m^2^. An abnormal ALT value (>40 U/L) was observed in 30 patients (35.2 %; 12 females). ALT values above 80 U/L were found in 13 patients (15.2 %; 4 females). Hepatitis C virus seropositivity (HCVab and HCV-RNA) was present respectively in 91% and 45.6% of TM patients (Table 1).

All the liver specimens showed at least grade 1 haemosiderosis; grades 3 and 4 occurred in seven out of 20 (35 %) with LIC levels from 16.5 to 21.3 mg/g/dw.

The median LIC was 2.4 mg/g dry weight (range: 0.1–24.6 mg/g dry weight). Six samples (7 %) had LIC over 15 mg/g/dw, a concentration associated with a high risk for cardiac disease^9^.

Total LIC levels correlated significantly with serum ferritin concentrations both in males and females (r: 0.724 and 0.65 respectively, p <0.01).

Forty TM patients (47%) had IGF-1 levels below the 2.5^th^ percentile of the normal values for the Italian population 8. No correlation was observed, in males and females, between IGF 1 values and LIC levels (r: 0.01, p: ns; r: 0.162, p: ns, respectively). Significantly lower IGF-1 levels were observed among those with liver cirrhosis (1 male patient) and severe stage of fibrosis (S=3–4, in 4 TM patients) according to the METAVIR Cooperative Study Group 4.

Seven TM patients (8.2%) with serum ferritin below 1500 ng/ml and LIC between 0.8 – 3.1 mg/g dry weight had very low IGF-1 levels (< 30 ng/ml). Three out of 7 had insulin dependent diabetes mellitus. Unfortunately, we have no data on growth hormone (GH)- IGF -1 axis in these patients.

There was also a positive correlation between serum ALT concentrations and LIC levels in males (r: 0.316; p < 0.05) and between serum ϒGT concentrations and LIC levels in females (r: 0.315; p < 0.05).

We acknowledge some limitation to our analysis. The relative small number of patients submitted to liver histology, the absence of data regarding the assessment of GH-IGF-1 axis and the effects of other associated endocrine complications, and the absence of information on the influence of other non-hepatic factors (smoking, alcohol intake), although they seem to be not relevant in our patients.

In summary, in the present study a significant correlation was observed between LIC and serum ferritin in all patients as well as between LIC and serum ALT concentrations in males and serum ϒGT concentrations in females. Furthermore, an association between severity of liver dysfunction and low IGF-1 levels was observed.

The gold standard for assessing liver iron stores, in the absence of cirrhosis, is the hepatic iron content determined by liver biopsy and quantitation with atomic absorption spectrophotometry 10. However, the use of biopsy-measured LIC as a marker of iron overload is limited by the small but finite risk of complications of liver biopsy, lack of reproducibility of quantitative assays, and sampling error 5. Non-invasive methods include blood tests (serum ferritin and iron saturation) and imaging techniques (MRI) or SQUID. Although there was a correlation between serum ferritin and LIC in this study, Li et al have shown that this correlation is less reliable at ferritin concentrations above 2500 ng/ml 10. MRI has been validated as a reliable non-invasive mean to assess iron stores in the liver, and the heart and SQUID has shown significant correlation with hepatic iron content as measured by biopsy 11, 12.

In conclusion, we believe that our data contribute further to the understanding of serum IGF-1 levels in TM patients and may represent a starting point for future studies for investigating the correlations between IGF-1, liver iron stores and severity of liver histology findings.

## Figures and Tables

**Table 1:** Demographic, clinical and laboratory features in 85 thalassaemia major patients

	Total study population of TM patients
Females/males (No.)	42/43
Mean age at the last observation/Age range	36.6 years/15.1–53.1 years
Body mass index (BMI, kg/m^2^) (Range)	17.4 – 30.8 kg/m^2^
ALT value >40 U/L) (No.; % )	30 patients (35.2 %; 12 females)
ALT value above 80 U/L (No.; % )	13 patients (15.2 %; 4 females).
Hepatitis C virus seropositivity (HCVab and HCV-RNA) (%)	91% and 45.6%
LIC over 15 mg/g/dw (% )	7%
IGF-1 levels < 2.5^th^ percentile of the normal values for the Italian population (%)	47 %
